# Glaucomatous changes in lamina pores shape within the lamina cribrosa using wide bandwidth, femtosecond mode-locked laser OCT

**DOI:** 10.1371/journal.pone.0181675

**Published:** 2017-07-25

**Authors:** Takuhei Shoji, Hiroto Kuroda, Masayuki Suzuki, Hisashi Ibuki, Makoto Araie, Shin Yoneya

**Affiliations:** 1 Department of Ophthalmology, Saitama Medical University, Iruma, Saitama, Japan; 2 Advanced Laser Medical Center, Department of Ophthalmology, Saitama Medical University, Iruma, Saitama, Japan; 3 Department of Ophthalmology, Kanto Central Hospital, Tokyo, Japan; Bascom Palmer Eye Institute, UNITED STATES

## Abstract

**Purpose:**

The lamina cribrosa (LC) is known to play a critical role in the pathogenesis of glaucoma. Although it has been reported that striae-shaped or slit-shaped lamina pores are more frequent in eyes with primary open angle glaucoma (POAG), this observation is based only on fundus photography. The primary object of this study is to perform layer-by-layer comparisons of the shape of lamina pores within the LC in vivo.

**Design:**

Cross-sectional study.

**Methods:**

Optic nerve head B-scans were obtained using custom-made broad-wavelength optical coherence tomography with a mode-locked laser. A total of 300 single B-scans per eye were obtained and three-dimensional images were rendered from these image sequences to obtain 2-μm thin-slice *en face* images of the LC. Elongation indices (EIs) of the lamina pores were measured from the anterior surface (AS) of the LC to the deeper layers in 40-μm increments.

**Results:**

Thirteen eyes from 10 primary open angle glaucoma (POAG) patients of mean deviation -15.2 (-16.5, -12.9) (median [25,75 percentile]) dB and 10 eyes from 7 normal controls were studied. Although the EI value was not significantly different between the superior, temporal and inferior regions of the LC at any depth level in either group, it was greater at the AS than at the 40 μm and 80 μm depth levels (*P* < .001) in both groups, and was greater in the POAG group only at the AS and 40 μm depth level (*P* ≤ .05). After adjustment for age and refraction, the effects of depth and presence of POAG on the EI value remained significant. Also, the severity of glaucoma and depth were significant factors associated with EI in multivariate analysis.

**Conclusions:**

Elongation of lamina pores was significantly more evident at the anterior surface and the 40-μm depth level of the LC in POAG eyes than in normal eyes, suggesting that nerve fiber bundles passing through the LC were under greater stress in the anterior layers of the LC.

## Introduction

The lamina cribrosa (LC) is a porous connective tissue, through which the axon bundles of retinal ganglion cells travel in transit to the orbital portion of the optic nerve. The LC is known to play a critical role in the pathogenesis of glaucoma.[1–3] Histopathological studies have reported various changes in the structure of the LC, such as thinning, posterior displacement, and decreased density of connective tissue, which are believed to be associated with key mechanisms underlying the retinal nerve fiber damage observed in glaucoma.[3–7] Miller et al. reported that striae-shaped or slit-shaped lamina pores were more frequent in eyes with advanced field loss.[8] However, this observation was mainly based on ophthalmoscopic observations or fundus photography, and the precise shape of the lamina pores within and on the surface of the LC was not determined. Moreover, the LC is partially hidden behind the converging retinal nerve fibers, and only a small portion is generally visible at the base of the optic cup in fundus photographs of normal eyes. Nerve fiber bundles passes through the lamina pores, the shape of laminar pores within LC may also play an important role in the pathogenesis of glaucoma.

Optical coherence tomography (OCT) is a noninvasive optical imaging modality that allows structural imaging of the fundus in patients. The development of spectral-domain (SD) detection technology has enabled production of three-dimensional (3D) images with high resolution. This method can not only provide detailed examinations of the retina, but also the choroid and deeper optic nerve head (ONH) structures, including the LC.[9, 10] Recent studies using SD-OCT devices revealed structural and dimensional changes in the LC associated with glaucomatous change, such as thinning, and posterior displacement of the LC and its reversal after a decrease in the intraocular pressure (IOP).[11–16] Any deformation of pores would imply that the nerve fiber bundles passing through these are also deformed, which may correlate with vulnerability of nerve fiber bundles to the chemical and/or mechanical damage that results in glaucomatous optic neuropathy.

To our knowledge, however, no studies to date have used OCT for layer-by-layer analysis in the LC. A high-resolution OCT system, based on a 200 nm bandwidth spectrometer and an 8-femtosecond ultrashort, mode-locked (ML), coherent laser light source, enabled in vivo cross-sectional ONH imaging with a 2.0 μm axial resolution and shorter raster scan interval resulting in a greater lateral resolution.[17, 18] Furthermore, the use of an ML coherent laser light source should allow us to obtain greater imaging depth and thin-slice *en face* imaging in the ONH than in the superluminescent diode light source, which is not a coherent light source in the strict sense.[17, 19]

Using this system, which should be more suited for thin-slice LC imaging than existing commercially available spectral-domain (SD)-OCT instruments,[19] we conducted layer-by-layer analysis of the shape of the lamina pores within the LC to determine any potential correlation in the observed changes with glaucoma.

## Materials and methods

The Ethics Committee of Saitama Medical University approved this cross-sectional comparative study, which was conducted in accordance with the tenets of the Declaration of Helsinki. Patients were included if they were at least 20 years old, fulfilled the eligibility requirements detailed below, and signed an informed consent form between April 2012 and July 2012. All subjects underwent complete ocular examinations. The Humphrey Field Analyzer 30–2 SITA standard program (HFA; Carl Zeiss Meditec, Inc., Dublin, CA) was used for visual field (VF) tests.

### Inclusion criteria

Only subjects with gonioscopically open angles were included. Patients with a diagnosis of primary open angle glaucoma [POAG], including normal tension glaucoma [NTG], that fulfilled the following criteria were considered eligible: characteristic glaucomatous ONH damage, including vertical cup-disc asymmetry between fellow eyes of ≥ 0.2 with neuroretinal rim damage, excavation, rim thinning, and notches with or without peripapillary hemorrhage; or retinal nerve fiber layer (RNFL) defects with a reproducible VF defect, including two or more contiguous points with a pattern deviation sensitivity loss of *P* < .01, three or more contiguous points with a sensitivity loss of *P* < .05 in the superior or inferior arcuate areas, or a 10 dB difference across the nasal horizontal midline at two or more adjacent locations and an abnormal result in the glaucoma hemifield test. Healthy volunteers aged at least 20 years were included as controls. The inclusion criteria for the control group were as follows: IOP <21 mmHg, reliable HFA results, absence of abnormal HFA findings suggestive of glaucoma in accordance with the criteria of Anderson and Patella,[20] absence of any apparent retinal disease, absence of glaucomatous optic neuropathy, and absence of any systemic and ophthalmological medication. Two independent masked glaucoma specialists (TS and MA) evaluated the normal appearance of the optic disc. In cases of disagreement, only eyes with discs that were unanimously determined as normal were included.

### Exclusion criteria

The exclusion criteria were as follows: visual acuity worse than 20/40; poor reliability of VF results (>20% fixation loss or >15% false-positive results); presence of any other ophthalmic disease, including media opacity, diabetic retinopathy, neuro-ophthalmological disease, uveitis, ocular trauma, and retinal or choroidal disease; presence of another disease capable of causing VF loss or optic nerve deterioration; presence of ocular treatment history with the exception of glaucoma medication; and a history of intraocular surgery or laser treatment.

### Instruments

**Fig 1:**
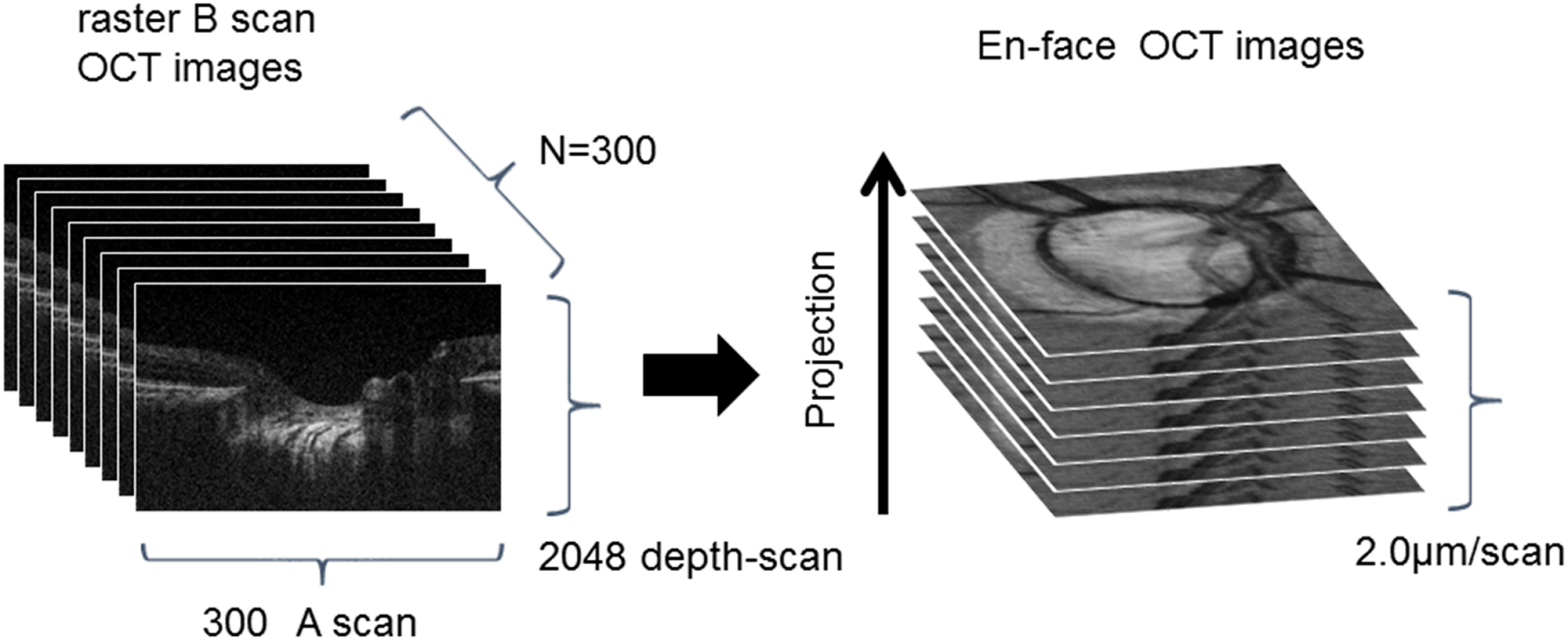
Diagram depicting the creation of an *en face* optic disc image from A-scan and B-scan images obtained with the optical coherence tomography device.

Fig 1 shows an OCT fundus image obtained by intensity integration, together with single scans obtained *in vivo*. We acquired a 3D image that visualized the connective tissue in the deep layers of the LC with a multilaminar sheet structure. The OCT system was built by the Advanced Laser Medical Center at Saitama Medical University. Details of the current SD-OCT system have been described elsewhere.[17–19, 21, 22] In brief, it is an OCT system using an ultra-broadband Kerr lens ML Ti:Sapphire laser and wideband spectrometer. The spectral bandwidth of the light source was 200 nm full-width at half maximum (FWHM) at a central wavelength of 840 nm. A high-speed charge-coupled device camera with 2048 × 300 pixels (Basler, Ahrensburg, Germany) was used as the detection system. The measurement speed was 50,000 depth scans/s, and the depth resolution was measured as <2.0 μm into the tissue.[17] The interferometer was attached to a semi-customized fundus scanning head system.

### Acquisition of *in vivo* 3D OCT images

OCT images were acquired after pupil dilation with tropicamide (Mydrin P; Santen, Osaka, Japan). The optic disc was also imaged using a digital 30° fundus camera (Zeiss FF450, Carl Zeiss, Jena, Germany) immediately before acquisition of the OCT data.

A raster scanning protocol with 300 B-scans and 300 A-scans (with 2048 pixels/A-scan) covering a 3.0 × 3.0 mm square region centered at the ONH was used for volumetric scans. Volumetric rendering of the 3D-OCT data set was performed, and *en face* cross sections were constructed using image processing software (Amira 5.4.3, Mercury Computer Systems Inc., Chelmsford, MA). A fundus image was generated as an *en face* projection image from the 3D data set by integrating the magnitudes of the OCT signals at each lateral position along the axial direction. The total data acquisition time for a single 3D-OCT (volumetric) image was 3.0 seconds. Small eye movements during the 3.0 second data acquisition were also adjusted by means of the image processing software (Amira).

### Assessment of lamina pores

**Fig 2:**
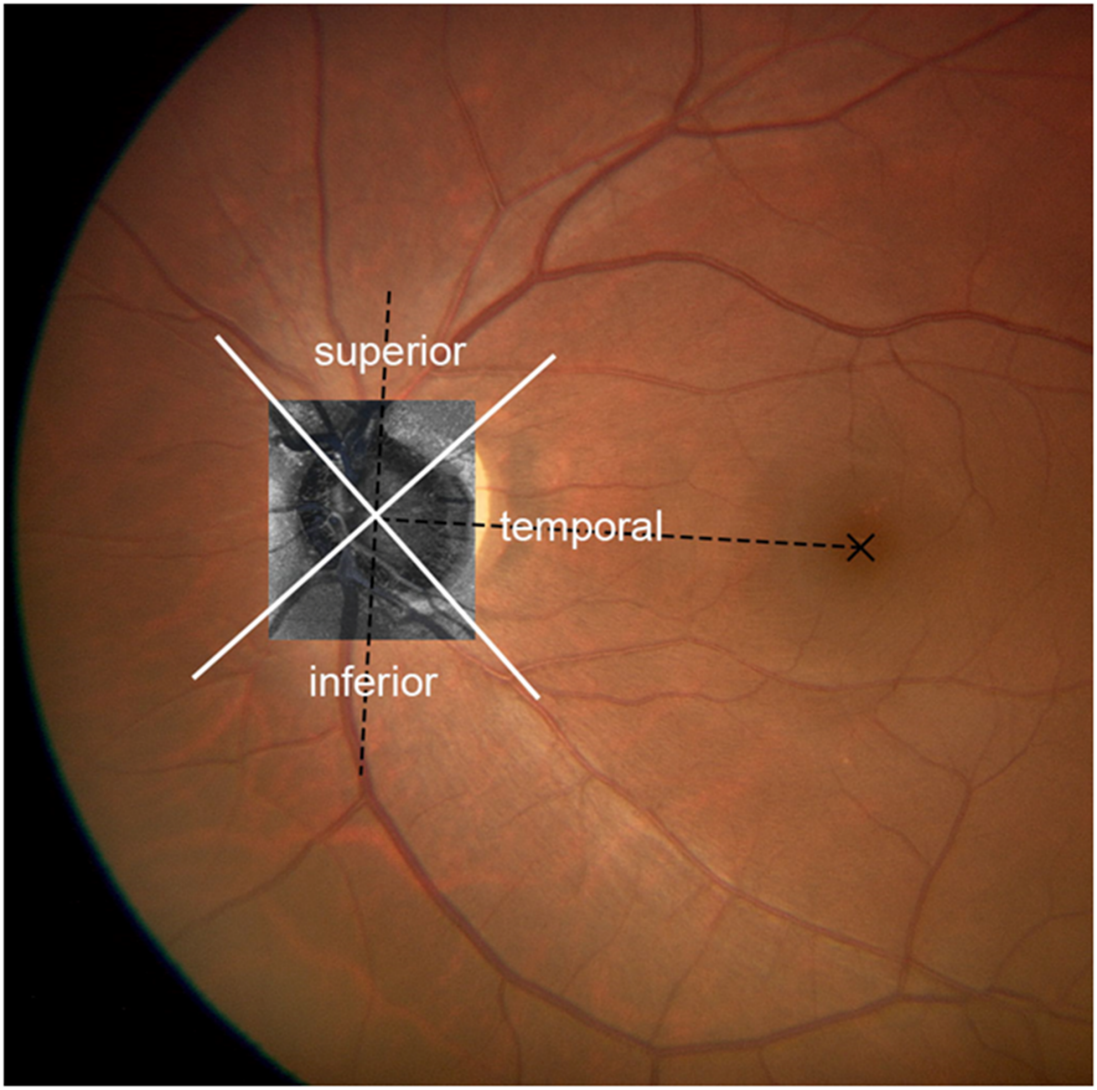
Schematic diagram of the 3 measurement regions in a left eye. The dotted line connecting the centroid of the disc margin and the fovea was designated as the reference line. A measurement sector at 0° to ±45° relative to the fovea center of disc axis was defined as the temporal region and the 45° to 135° circumferentially superior and inferior directions as the superior and inferior regions.

After acquisition of an *in vivo* 3D dataset, Bruch membrane opening (BMO) was detected and marked as reported previously, [21] and the AS borders of the LC were considered to be where the highly reflective region started within the ONH on the basis of previous studies.[14, 23] The AS plane of the LC was defined as the lowest AS lines using simultaneous visualization of 3 modalities (color, B scan, and en face images) to increase the precision in identifying the AS of the LC plane by two investigators (MA, TS) who have expertise in inspecting images obtained with using current system. We compensated for tilting in the event of a significantly tilted LC based on the AS line. The LC area was divided into 3 regions (Fig 2).[24] A measurement sector at 0° to ±45° relative to the fovea center of the BMO axis was defined as the temporal region and the 45° to 135° circumferentially superior and inferior directions as superior and inferior regions, respectively. The center of gravity of the BMO[21] and the center of the fovea were marked by hand on the fundus photographs which were superimposed on the en face projection images.

**Fig 3:**
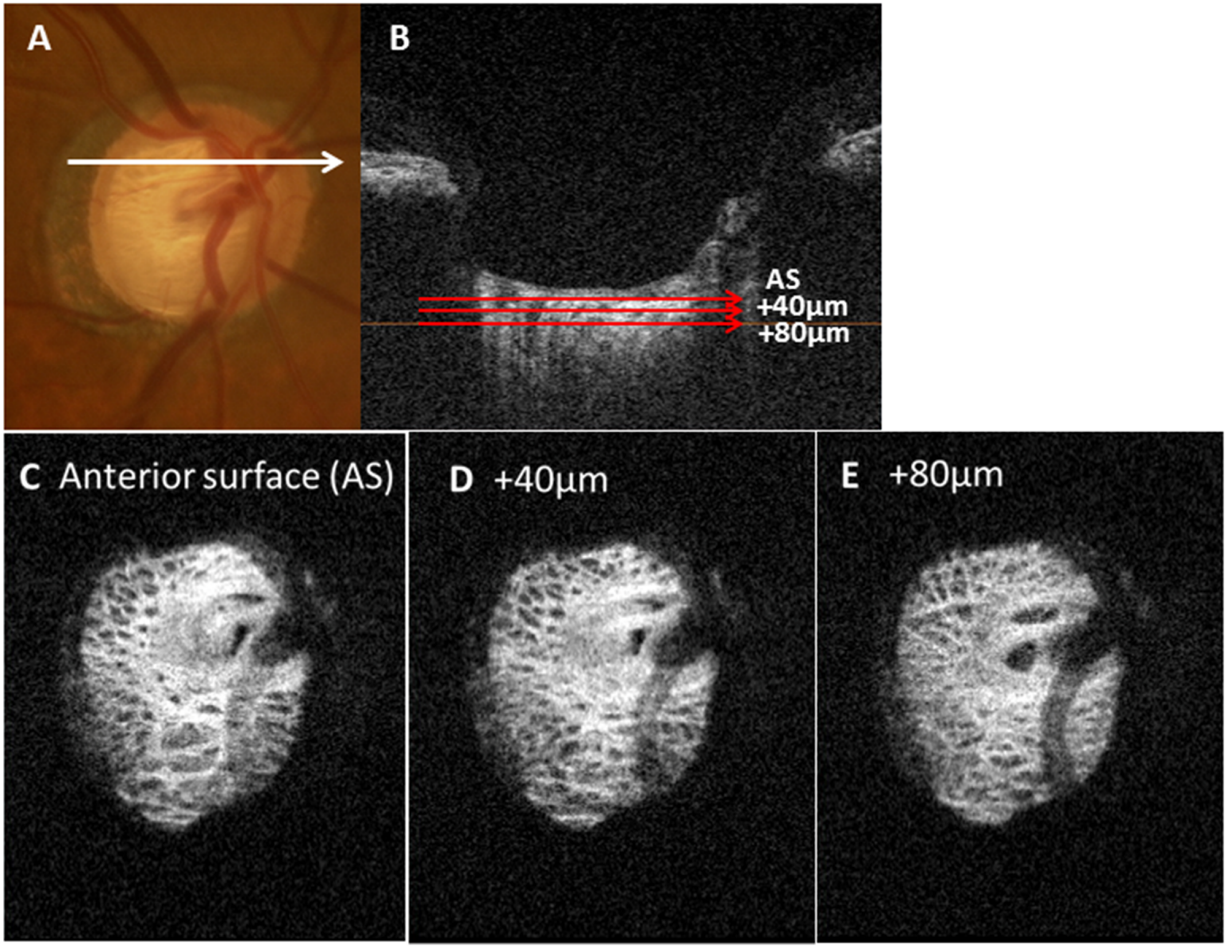
Schematic explanation of the method of acquisition of en face images. (A) Photographic image of a fundus (B) The anterior surface (AS) of the lamina cribrosa (LC) was determined from a B-scan image. Depths of 40 μm and 80 μm were determined from the AS images. (C–E) Each en face image of the AS of the LC (C), 40 μm from the AS (D) and 80μm from the AS (E).

The depth levels of the LC currently studied were the AS, a depth of 40 μm from the AS along the z-axis (40 μm depth level) and a depth of 80 μm from the AS (80 μm depth level). Since two independent examiners (ST, SY) agreed that the LC pores became difficult to demarcate reliably at a depth of 120 μm in 7 of 23 eyes (30%), the analyses were performed at the AS and depth levels of 40 μm and 80 μm (Fig 3).

**Fig 4:**
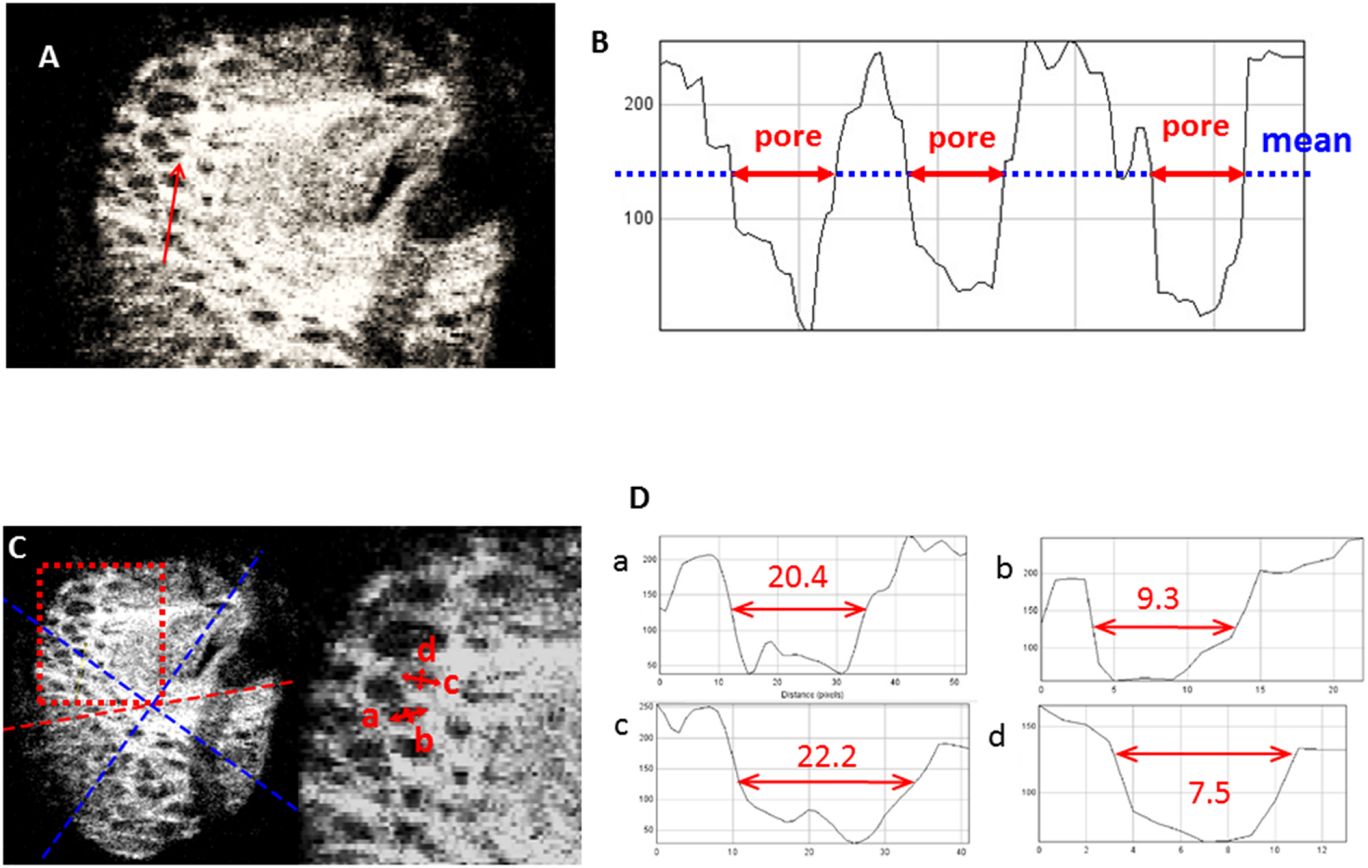
Schematic explanation of the method for measuring the elongation index (EI) for the lamina pores in the lamina cribrosa (LC). (A) En face image and line scan (red arrow). (B) The reflectivity of the lamina pore is tabulated using the Image J program. The boundary of the lamina pore (red arrow) was defined as the point where its reflectivity is lower than the mean lamina beam reflectivity (blue dot line). (C) En face image (left) and magnified view (right). Red dot square corresponds to a magnified view. The red dot line is the fovea-disc line and blue dot line divides each region. (D) Representative pore analysis. In this case, the longest diameter is 20.4 pixels (a) and the shortest diameter is 9.3 pixels (b) for one pore (red arrows), which means that the EI is 2.19. For another pore, the longest diameter is 22.2 pixels (c) and the shortest diameter is 7.5 pixels (red arrows) (d), which means that the EI is 2.96.

The reflectivity of the lamina pore was measured using Image J software (ver. 1.43, developed by Wayne Rasband, National Institutes of Health, Bethesda, MD, http://rsb.info.nih.gov//ij) with the plot profile function (Fig 4) to avoid subjective visual determination of the margins of the lamina pores. The mean reflectivity of each across the entire en face LC image was measured and its margins were defined on grayscale images as the series of points at which the reflectivity diminished to below the mean reflectivity of each entire en face image. This method using the plot profile function was also adopted in a previous study to objectively determine photoreceptor inner/outer segment (IS/OS) defect margins on OCT images.[25]

Two masked examiners blinded to any information other than the laminar pore images, examiner-1 (HI) and -2 (MS), identified all laminar pores for which the margins were thought to be measurable using the above method according to each examiner’s discretion at each of the three regions (superior, temporal, or inferior) and LC depth level (AS, 40 μm and 80 μm-depth levels). The two examiners independently determined the EI, the reciprocal of the ovality index, that is, the major axis length/minor axis length, [26] at each specific region and depth for each subject eye, and the median of the EI values was adopted by examiner-1 EI and examiner-2 EI at each specific region and depth, respectively. The mean of the above median EIs given by the two examiners was adopted as the final EI value at each specific region and depth of each subject eye to be used for further analysis.

To evaluate the reproducibility of the current method used to measure the EI value, 54 en face OCT images of various regions and depths were extracted and evaluated twice at intervals of 2 days by the same pair of examiners (examiner-1 and -2) to calculate an intraclass correlation coefficient [ICC (1,1)] of the EI value. To evaluate inter-examiner group reproducibility, the same en face OCT images were independently evaluated by another pair of blinded examiners (examiner-3 and examiner-4; HK and SY). The obtained EI values and those obtained by the previous group of examiners (examiner-1 and examiner-2) were used to calculate the ICC (2,1).

#### Commercially available spectral-domain OCT imaging

All subjects also completed at para-papillary (Spectralis HRAþOCT; Heidelberg Engineering, Heidelberg,Germany). The circumpapillary RNFLT (cpRNFL) was measured with the Spectralis SD-OCT parapapillary circle scan (software version 5.4.7.0). The examiner is required to place the scan manually around the optic disc at the baseline examination. After the reference image is identified manually by the operator, the system recognizes the reference image scanning area and automatically positions the retest scan on the same location in followup examinations. The scan circle contains 1536 A-scan points from a 12° circle, which equates to a retinal diameter of 3.5 mm in eyes with standard corneal curvature. The acquisition rate is 40 000 A-scans per second.

### Statistical analyses

The data are expressed as the mean ± standard deviation (SD) for continuous variables and frequencies (percentages) for categorical variables. The age, MD and EI were not normally distributed using the Shapiro–Wilk W test. So these parameters were expressed as the median (25,75 percentile) and compared using the nonparametric Mann–Whitney U test or Kruskal–Wallis test as appropriate. Bilateral eyes were included in the analyses if they matched the inclusion criteria. The null hypothesis was that the EI of each region and each depth would not differ significantly between groups. The alternative hypothesis was that the EI would differ significantly between groups. To determine the required sample size, we assumed that mean EI value would be 1.4 in control group and 2.0 in glaucoma group with estimated standard deviation of 0.3. A sample size of 16 eyes would provide 80% power to detect a clinically significant difference between the groups using a 2-sided test at a 5% significance level. Because measurements from both eyes of the same subject are likely to be correlated, the Generalized Estimating Equation (GEE) was used to analyze data derived from such eyes.[27] The GEE method was also used to adjust for within-individual repeated measurements correlation[28] and to evaluate the mean difference between groups.[29, 30] To determine the effects of various factors on the EI values, we performed crude, age-adjusted, and multivariate analyses with GEE. Model 1 was adjusted for age (decades), IOP (mmHg), POAG (presence or absence), region (superior, inferior, temporal), depth (at AS and depth levels of 40 μm and at 80 μm), and refractive error (dioptres). Model 2 was adjusted for age (decades), IOP (mmHg), MD (dB), region, depth, and refractive error (dioptres). Model 3 was adjusted for age (decades), IOP (mmHg), cpRNFL thickness (μm), region, depth, and refractive error (dioptres). To avoid problems of multicollinearity, the multivariate analysis was performed in several ways when there were factors correlated with each other. A *P*-value of <0.05 was considered to be statistically significant. All statistical analyses were performed using JMP version 10.1 software (SAS Institute, Inc., Cary, NC, USA) and SPSS version 22 software (Japan IBM, Tokyo, Japan).

## Results

Twenty-six eyes of 19 participants were initially enrolled, of which 3 eyes were excluded because of incorrect OCT images attributable to inappropriate fixation, leaving 23 eyes (13 eyes with POAG in 10 patients, including 11 eyes with NTG in 8 patients, and 10 normal eyes in 7 subjects) for analysis. All glaucoma patients was taking one or more topical glaucoma medications.

A total of 1112 pores were measured for calculating EI, and median value at each region and at each depth was analyzed. Table 1 summarizes the baseline characteristics of the study subjects. While the mean spherical equivalent was not significantly different between the two groups, the mean deviation (MD) value was significantly worse and the patient age was significantly older in the POAG group than in the control group.

**Table 1:** Baseline characteristics of study population.

	Control group	POAG group	P value
**By subject no**.	7	10	
Male (n, %)	3 (42.9)	7 (70.0)	0.350*
Age (yrs) (median[25,75 percentile])	30 (30, 40)	67 (56, 72)	<0.001^†^
**By Eye no**.	10	13	
Spherical equivalent error (D)	-1.4±1.5	-1.9±3.3	0.685^‡^
IOP (mmHg)	14.3±2.0	16.7±1.9	0.009^‡^
cpRNFL thickness (μm) (median[25,75 percentile])	98 (90, 106)	57 (44, 63)	<0.001^†^
MD (dB) (median[25,75 percentile])	0.1 (-0.3, 0.3)	-15.2 (-16.5, -12.9)	<0.001^†^

Plus-minus values are means ± SD.

*Chi-square test,

^†^Mann–Whitney U test,

^‡^Unpaired t-test

Abbreviations: POAG, primary open angle glaucoma; yrs, years; IOP, intraocular pressure; cpRNFL, circumpapillary retinal nerve fiber layer; MD, mean deviation; dB, decibels; D, diopters

**Fig 5:**
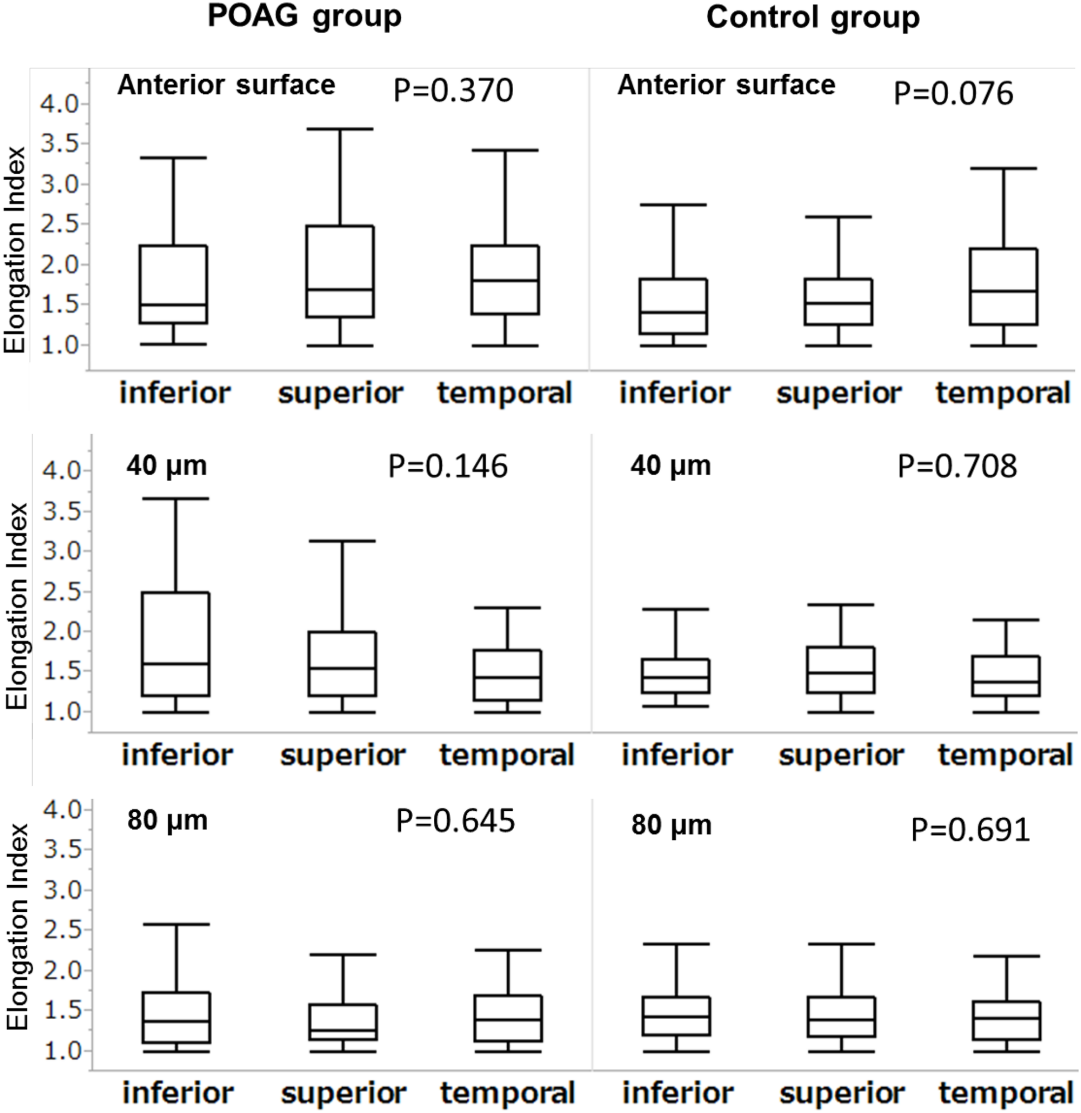
Box plot for the elongation index (EI) and region analysis.

The EI measurements showed good intra-examiner group and inter-examiner group reproducibility (ICC[1,1] = 0.935 and ICC[2,1] = 0.897 [95% confidence intervals (CIs) 0.889–0.962 and 0.829–0.939], respectively, *P* < .001). Fig 5 shows a box plot for all the EI values obtained for each region at each depth level. Although the EI values tended to be larger in the superior region at the anterior surface (AS) level in the POAG group, there was no significant difference in the EI value between the 3 regions in either the control group or POAG group at any depth level.

**Fig 6:**
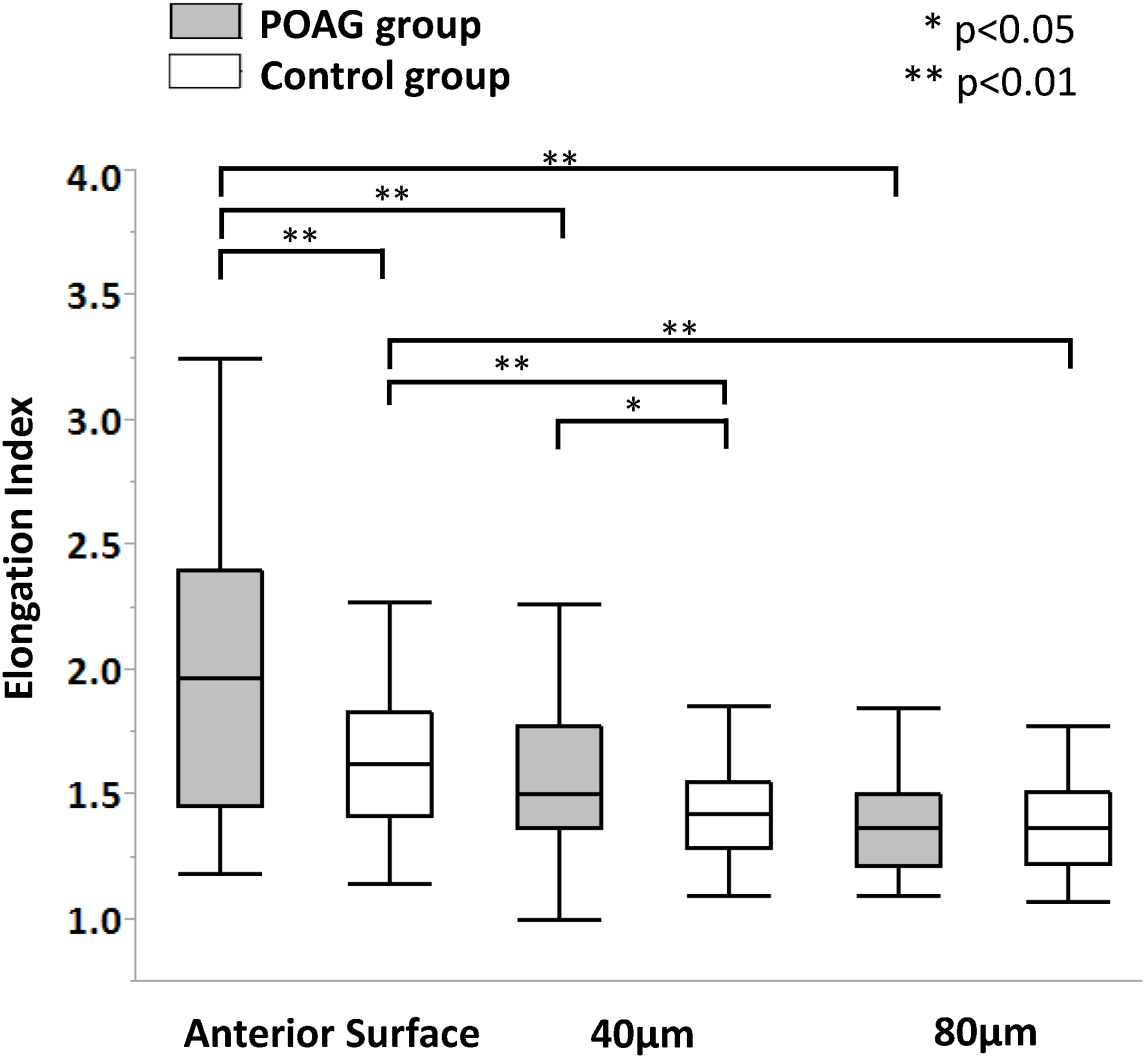
Box plot for the elongation index (EI) and depth analysis.

Since the EI values were not significantly different between the 3 regions at any depth level in either group, they were further averaged for each depth level for inter-group and inter-depth comparison. Fig 6 shows a box plot for the EI values at each depth level in both groups.

The median (25,75 percentile) EI value was 1.96 (1.45, 2.40) and 1.63 (1.42, 1.83) at the AS, 1.50 (1.36, 1.78) and 1.42 (1.29, 1.55) at the 40 μm depth level, and 1.37 (1.21, 1.50) and 1.37 (1.22, 1.51) at the 80 μm depth level in the POAG (n = 13) and control groups (n = 10), respectively (Table 2). The EI values at the AS were significantly greater than those at the 40 μm and 80 μm depth level in both groups (*P* < .001). Moreover, the EI values at the AS and 40 μm depth in the POAG group were significantly larger those in the control group (*P* = .005 and *P* = .046, respectively).

**Table 2:** Lamina pore elongation index in each group.

	*Elongation Index (Median [25-*,*75-pecentile])*				
*Variables*	*Control group*	P value*	*POAG group*	P value*	*P value*^†^
***Depth***					
***Anterior surface of LC***	1.63 (1.42, 1.83)		1.96 (1.45, 2.40)		**0.005**
***40μm depth***	1.42 (1.29, 1.55)	**<0.001**	1.50 (1.36, 1.78)	**<0.001**	**0.046**
***80μm depth***	1.37 (1.22, 1.51)	**<0.001**	1.37 (1.21, 1.50)	**<0.001**	0.942

Abbreviations: LC, lamina cribrosa; POAG, primary open angle glaucoma

* P value for Steel’s test compared to anterior surface of LC

^†^ P value for Control group vs. POAG group using Mann–Whitney U test

Table 3 shows the results of crude, age-adjusted, and multivariate analyses for factors potentially affecting the EI value. Model 1 was adjusted for age (in decades), gender, POAG (presence or absence), region (superior, inferior, and temporal), depth (at the AS and depth levels of 40 μm and 80 μm), and refractive errors. Model 2 was adjusted for age (in decades), gender, MD (dB), region, depth, and refractive errors. Model 3 was adjusted for age (in decades), gender, IOP, cpRNFL thickness, location, depth, and refractive errors. Model 1 and 2 showed that POAG (*P* = .0013), MD (*P* < .0001), and the AS layer (*P* < .001) remained as significant contributing factors after adjustment for other potential confounding factors, indicating that the difference in the EI value between POAG and normal control eyes was significant after adjustment for age or refraction. Also, the *P*-value for trend of depth was significant (*P* < .001), which confirmed that deeper layers were associated with smaller EI values.

**Table 3:** Association of potential clinical and biometric parameters with the elongation index based on univariate and multivariate analyses.

	*Elongation Index*									
	*Crude*		*Age Adjusted*		*Multivariate (Model 1)*		*Multivariate (Model 2)*		*Multivariate (Model 3)*	
*Variables*	*Coefficients (95%CI)*	*P Value*	*Coefficients (95%CI)*	*P Value*	*Coefficients (95%CI)*	*P Value*	*Coefficients (95%CI)*	*P Value*	*Coefficients (95%CI)*	*P Value*
***Age (per decades)***	0.05 (0.01, 0.10)	**0.029**			−0.05 (−0.14, 0.04)	0.298	−0.02 (−0.05, 0.12)	0.330	0.02 (−0.06, 0.09)	0.675
***Gender (reference*: *male)***	-0.07 (−0.24, 0.11)	0.450	-0.00 (−0.14, 0.13)	0.948	0.03 (−0.09, 0.15)	0.622	-0.00 (−0.10, 0.09)	0.937	-0.06 (−0.13, 0.26)	0.533
***SEQ***	0.02 (−0.01, 0.05)	0.294	0.01 (−0.02, 0.05)	0.355	0.02 (−0.01, 0.05)	0.234	0.00 (−0.02, 0.02)	0.886	0.01 (−0.03, 0.04)	0.652
***IOP (per mmHg)***	0.01 (-0.02, 0.03)	0.732	-0.01 (-0.03, 0.02)	0.600	-0.00 (-0.01, 0.00)	0.360	-0.00 (-0.01, 0.00)	0.400	-0.01 (-0.01, 0.00)	0.456
***POAG (reference*: *control)***	0.21 (0.07, 0.36)	**0.004**	0.23 (0.05, 0.41)	**0.013**	0.43 (0.14, 0.71)	**0.003**				
***MD (per dB)***	-0.02 (-0.03, -0.02)	**<0.001**	-0.03 (-0.03, -0.02)	**<0.001**			-0.03 (-0.03, -0.02)	**<0.001**		
***cpRNFL (per 10μm)***	-0.04 (-0.06, -0.01)	**0.009**	-0.02 (-0.06, 0.02)	0.276					-0.03 (-0.08, 0.01)	0.154
***Location (reference*: *temporal)***										
***superior***	0.08 (−0.03, 0.19)	0.153	0.08 (−0.03, 0.19)	0.150	0.08 (−0.03, 0.19)	0.157	0.08 (−0.03, 0.19)	0.154	0.08 (−0.03, 0.19)	0.154
***Inferior***	−0.03 (−0.13, 0.07)	0.505	−0.04 (−0.14, 0.07)	0.494	−0.03 (−0.13, 0.07)	0.514	−0.03 (−0.13, 0.07)	0.508	−0.03 (−0.13, 0.07)	0.503
***Depth (reference*: *AS)***										
***40μm***	−0.32 (-0.46, −0.19)	**<0.001**	−0.33 (−0.46, −0.19)	**<0.001**	−0.32 (−0.46, −0.19)	**<0.001**	−0.32 (−0.46, −0.19)	**<0.001**	−0.32 (−0.46, −0.19)	**<0.001**
***80 μm***	−0.47 (−0.67, −0.28)	**<0.001**	−0.48 (−0.67, −0.27)	**<0.001**	−0.47 (−0.67, −0.27)	**<0.001**	−0.47 (−0.67, −0.27)	**<0.001**	−0.48 (−0.68, −0.27)	**<0.001**
***p for trend***		**<0.001**		**<0.001**		**<0.001**		**<0.001**		**<0.001**

**Abbreviations**: SEQ, spherical equivalent; IOP, intraocular pressure; POAG, primary open angle glaucoma;MD, mean deviation; cpRNFL, circumpapillary retinal nerve fiber layer; dB, decibels; D, dioptres; AS, anterior surface

Model 1 is adjusted for age, gender, SEQ, IOP, presence of POAG, location, and depth

Model 2 is adjusted for age, gender, SEQ, IOP, MD, location, and depth

Model 3 is adjusted for age, gender, SEQ, IOP, cpRNFL thickness, location, and depth

## Discussion

The principal finding of this study is that elongation of lamina pores (greater EIs) was more evident at the AS than at depth levels of 40 μm and 80 μm. The changes were depth-dependent in both the POAG group and the normal group, and those for POAG eyes were significantly greater at the AS and 40 μm depth level than those for normal control eyes. The presence of more elongated or deformed lamina pores in POAG eyes in the superficial layers of the LC implies that the nerve fiber bundles passing through these pores may be under more inhomogeneous stress. This stress may relate to the nerve fiber bundle impairment associated with glaucomatous optic neuropathy.

To the best of our knowledge, this is the first *in vivo* study to evaluate intra-eye layer-by-layer comparisons of lamina pore shape between POAG and normal eyes. Compared with a superluminescent diode light source with the spectral width of 60–100 nm used in the commercially available SD-OCT instruments, the current OCT method uses a femtosecond ML laser with a 200 nm homogeneous wavelength range as a light source. The theoretical and practical advantages of an ML laser when compared to a superluminescent diode light source in OCT have already been reported.[17, 19] The depth resolution of our OCT devices was found to be as high as 2.0 μm into the tissue with a shorter raster scan interval resulting in 2–4-fold higher lateral and depth resolution when compared with current OCT instruments, enabling acquisition of thin-slice images and more detailed layer-by-layer analysis of the LC.[17–19] Previous histological studies have revealed that the surface and pores of the LC become deformed with progression of glaucoma.[8, 31] In addition, previous *in vivo* reports have shown that the lamina pores are more elongated in glaucomatous eyes than in normal eyes.[8, 26, 32, 33] These observations are consistent with our results for EI values at the AS and 40 μm depth level. Although scanning laser ophthalmoscopy (SLO) modalities, including AO-SLO, have been reported to be superior to fundus photography for visualization of the lamina pores,[26, 33, 34] SLO imaging might not be able to delineate lamina pores at the deeper layers of the lamina. The human LC is considered to be approximately 10 layers thick[35] and 175–345 μm in depth.[10, 14, 15] The current findings suggest that the pore elongation in POAG eyes is limited to the anterior layer (up to a depth level of 40 μm from the AS) of the LC, implying that deformation of the glaucomatous laminar pores is LC depth-dependent and the anterior layer is more important. Abnormal depth dependency of elongation or deformation of the lamina pore, rather than elongation itself, may be more crucial to the passing of axons through the LC. Glaucomatous axonal degeneration is related to morphological changes in the LC.[36] The evaluation of EI at different LC layers may provide useful information for extending our understanding of glaucoma-induced LC damage. In this study, EI values were correlated with both the presence of glaucoma and MD, but not with cpRNFL thickness. CpRNFL thickness is thought to be an early phase index to detect glaucoma because presence of characteristic visual field defects can confirm the diagnosis, but as many as 30% to 50%of retinal ganglion cells may be lost before defects are detectable by standard visual field testing. [37] Thus, the current results may suggest that EI change in glaucoma eyes are correlated with glaucoma functional progression rather than early phase glaucoma detection. Further larger sample size and longitudinal studies will be needed to confirm the relationship between progression of glaucoma and each depth pore elongation.

Histopathological studies in human eyes demonstrated greater compression and backward bowing in the superior and inferior regions of the LC when compared with the other regions in patients with glaucoma.[6, 7] Such regional differences in LC changes have been attributed to structure differences within the LC, such as the lower density of connective tissue and larger lamina pores in the superior and inferior regions. In this study, the inter-region difference in the LC value was statistically marginal (*p* = .076) only at the AS of the LC. *In vivo*, the lamina pores are comprised of not only collagen fibers but also blood vessels, extracellular matrix components (other than collagen fibers), and astroglia, which would be lost after trypsin digestion,[31] suggesting that the shape of the lamina pore observed in in vivo study is not always the same as that observed in histological studies. These differences may be at least partly responsible for differences between the results of *ex vivo* histological studies and those in the current study. The relatively small number of POAG eyes in this study, especially those with advanced damage, could be a possible explanation.

Several limitations of this study warrant discussion. First, this was a pilot study of custom-made OCT in a relatively small number of subjects. Differences in lamina pore dimensions may be influenced by a number of factors, including age, and axial length. In particular, the age between the groups was significantly different. This is translational research lying between applied physics and medicine using custom-made OCT with femtosecond mode-locked laser and is a preclinical study. The generalized estimating equation (GEE) approach has been used in previous ophthalmological OCT studies to adjust for differences between groups.[29, 38] Although the effect of age was not significant in multivariate analysis and the coefficient (-0.02 per decade) and 95% CI (-0.10 to 0.02 per decade) for age were far smaller than the other factors, the influence of aging should be investigated carefully. Moreover, whereas the location factor is not statistically significant in this study, the EI of location would be significant with larger sample size. Further studies in a larger number of subjects matched for age and various disease stages will be required to confirm the current findings. Second, we did not correct the magnification of our OCT images. According to Littman,[39] uncorrected lateral measurements tend to underestimate the true dimensions as the axial length increases. However, because the EI was described here using a short axis-long axis ratio, the impact of magnification effects is expected to be minimal. Third, we could not determine EI values at the 120 μm depth level in all subjects. An OCT instrument using an ML coherent laser light source with a longer central wavelength will provide a greater imaging depth than the currently available SS-OCT system.[40] Fourth, some eyes with glaucoma have posteriorly curved LC, which might underestimate EIs because posteriorly curved pores seem to be more round than actual and it is possible that our study might have underestimated EIs with glaucoma. However, the effect of this on the study results would be minimal because the EIs for eyes with glaucoma were significantly larger than those for normal eyes even though EIs in some eyes were underestimated. Fifth, the limitations of OCT technology produce shadows beneath the retinal vessels. The resulting high concentration of shadows could affect accurate assessment of the LC pores. Thus, we manually defined pores of the scanned image that could be reliably segmented, and only measured LC pores in this area. In the near future, enhancement techniques such as adaptive compensation may enable clearer visualization of the LC surface beneath the vessels or thick sections of the neuronal rim, thereby increasing the accuracy of measurements of LC pores.[41]

In conclusion, the current OCT system enabled *in vivo* intra-eye layer-by-layer comparisons of lamina pore shape. Elongation of lamina pores or an increase in the EI value was more evident in the anterior layer of the LC in the POAG eyes than in the normal control eyes, suggesting that nerve fiber bundles passing through the LC might be injured mainly in the anterior layer of the LC.

## Supporting information

S1 Video Stepwise demonstration of the measurement of lamina cribrosa elongation indices. (MP4)

S1 Dataset Results of lamina pore elongation index. (XLSX)
